# Identification and quantification of PFAS in the tap water of a French City, Besançon

**DOI:** 10.1007/s10661-026-15483-y

**Published:** 2026-05-23

**Authors:** Grégorio Crini, Chiara Mongioví, Corina Bradu, Nicolas Audonnet, Stephen Yquel, Dario Lacalamita

**Affiliations:** 1 Laboratoire Chrono-Environnement, Université Marie et Louis Pasteur, 16 Route de Gray, 25000 Besançon, France; 2 Istituto per i Processi Chimico-Fisici, CNR, Via E. Orabona 4, 70125 Bari, Italy; 3 Department of Systems Ecology and Sustainability, PROTMED Research Centre, University of Bucharest, Bucharest, Romania; 4 Laboratoire IANESCO, 6 Rue Carol Heitz, 86000 Poitiers, France; 5 Department of Chemistry, Università Degli Studi di Bari Aldo Moro, Via E. Orabona 4, 70125 Bari, Italy

**Keywords:** Perfluoroalkyl substances, Water policy, Trifluoroacetic acid, Tap water, Analytical monitoring, LC–MS/MS

## Abstract

In France, information on per- and poly-fluoroalkyl substances (PFAS) in residential tap water is still limited or non-existent. In this study, we conducted a local survey to compare human exposure to PFAS in 28 private family households in a medium-sized French city (Besançon). We present the results on the identification and quantification of 65 PFAS substances in residential tap water. Concentrations of PFAS were assessed by a French accredited laboratory. A total of 74 tap water samples were analyzed, revealing the presence of 12 individual molecules, 8 of which are among the 20 regulated substances. Forty-one samples contained a mixture of 2 to 10 different PFAS substances, the most frequently quantified molecules being trifluoroacetic acid (100%), perfluorooctanoic acid (48.6%), perfluorooctane sulfonic acid (44.6%), perfluorobutanoic acid (27.0%), perfluoropentanoic acid (50.1%), and perfluorohexanoic acid (23.0%). The corresponding cumulative concentrations (sum of 65 substances) ranged from 440 ng/L to 3,805 ng/L, showing significant variability. However, in terms of regulations, all values were below the 100 ng/L limit for the sum of the 20 molecules. Trifluoroacetic acid, which is not on the regulatory PFAS list, was systematically found in all the water samples analyzed and the results clearly highlighted the dominance of this PFAS. In tap water, trifluoroacetic acid was quantified at concentrations ranging from 440 to 3,800 ng/L (average value of 1096 ± 575 ng/L), *i.e.*, 4 to 38 times higher than the regulatory threshold of 100 ng/L. This study underlines the need to monitor not only the 20 substances listed in the European Directive, transposed into French law, but also other PFAS, and, above all, to include trifluoroacetic acid.

**Graphical Abstract:**

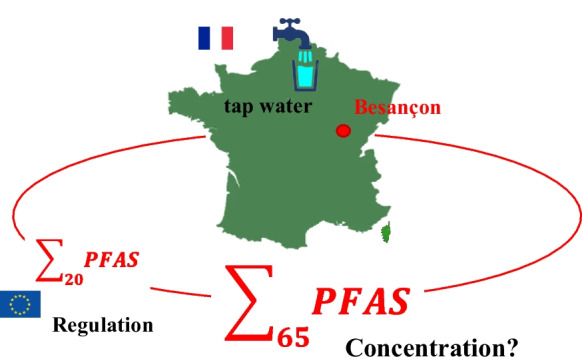

**Supplementary Information:**

The online version contains supplementary material available at 10.1007/s10661-026-15483-y.

## Introduction

Per- and polyfluoroalkyl substances (PFAS) are a broad family of fluorinated chemicals first synthesized in the 1930 s and widely used worldwide since the 1950s. According to Organisation for Economic Co-operation and Development (OECD), PFAS are fluorinated substances containing at least one fully fluorinated methyl (–CF_3_) or methylene (–CF_2_–) carbon atom. Their many, varied and unique properties – including high stability, heat resistance, non-adhesion, and impermeability – are commonly used in many industrial processes and common products (Cordner et al., 2024; Doria, 2006; et al.,e et al., 2018; Huber et al., 2024; Kemper et al., 2024; Li et al., 2025; Pezeshki et al., 2025; Smalling et al., 2023).

PFAS have been recognized as environmental contaminants of emerging concern because they do not degrade easily in the environment due to the carbon–fluorine bond, one of the strongest in chemistry. There is growing evidence that these substances, along with their metabolites, are present in ecosystems across the planet (Cordner et al., 2024; Pezeshki et al., 2025).

From a human perspective, several PFAS compounds have raised concern due to their endocrine-disrupting and carcinogenic properties (Göckener et al., 2020; Pezeshki et al., 2025; Shao et al., 2025; Cuchimaque Lugo et al., 2026; Pulster et al., 2026). A recent study on the exposure to PFAS in tap water in the United States has been associated with various cancers (Li et al., 2025). Several studies have linked exposure to certain legacy PFAS compounds through drinking water to adverse health outcomes, including increased cancer risk. While substantial scientific knowledge is available for well-studied PFAS such as PFOA, PFOS, and PFHxS, important knowledge gaps remain for many other PFAS, particularly emerging and ultrashort-chain compounds.

In Europe, PFOS, PFOA, and PFHxS were banned from production, use, and marketing in 2009, 2020, and 2022, respectively. Nevertheless, these compounds are still frequently detected in environmental and biological matrices due to their high persistence and the legacy of past emissions (Göckener et al., 2020). Some substances are found in surface and groundwater sources, and can accumulate in living organisms, both plants and animals, and end up in the food chain (Adewuyi & Li, 2024; Arp et al., 2024; Bai & Son, 2021; Cappelli et al., 2024; Cordner et al., 2024; Domingo & Nadal, 2019; Gaillard et al., 2024).

The quality and sustainability of drinking water at the point-of-use, as well as the protection of resources, are growing concerns in France, particularly in connection with the European policy on water. In 2020, the European Union (EU) issued a directive on the quality of water intended for human consumption (EU Directive, 2020/2184 of 16 December 2020), listing 20 PFAS substances, three of which are banned. This directive came into force in January 2026 in all EU member states. A quality limit of 100 ng/L has been set for the sum of the 20 molecules (Σ_20_ PFAS) described in this directive. It is important to point out that the simplest PFAS, trifluoroacetic acid (TFA), has not been included in this list. Another parameter, total PFAS (Σ_total_ PFAS), has also been introduced, with an associated quality limit of 500 ng/L, designed to integrate all measurable PFAS in water. The European directive was transposed into French law in January 2023 (French Official Journal, no. 0147, June 27, 2023).

In France, tap water is the most strictly controlled foodstuff, subject to ongoing health monitoring to guarantee its safety, from the source of extraction (catchment in the natural environment) to the consumer’s tap. Drinking-water exposures to PFAS are a recent national concern, but information on PFAS in residential tap water is limited. Although some initiatives have already started in 2023, systematic research on PFAS will only be integrated into the sanitary monitoring of water intended for human consumption by the *Agences Régionales de Santé* (Regional Health Agencies) starting from 12 January 2026. The chosen regulatory value to be respected will be 100 ng/L for the Σ_20_ PFAS. Furthermore, in France, exceeding a regulatory threshold does not automatically mean that water is unfit for consumption, as is the case for certain pesticides detected in tap water.

Analytical data on the identification and quantification of PFAS in drinking water are still lacking and need to be completed in order to reassure the public (Spyrakis & Dragani, 2023). This is one of the reasons why, on January 17, 2023, France launched a PFAS 2023–2027 action plan led by the *Ministère de la Transition écologique et la cohésion des territoires* (Ministry of Ecological Transition and Territorial Cohesion), designed to reduce exposure to these forever pollutants.

One year later, on April 5, 2024, this first plan was supplemented by an inter-ministerial action plan mobilizing all public administrations to reduce “as quickly as possible the risks associated with PFAS, particularly those linked to molecules present in water intended for human consumption” (French Ministry of Labor, Health, Solidarity and Families, 2024). This new plan is organized into 5 main areas, the first of which aims to better quantify and measure PFAS in tap water, groundwater, industrial discharges, and aquatic environments.

Within this framework, we investigated the occurrence of PFAS, including TFA, in tap and bottled water samples as part of the Franco–Italian–Romanian project WATEREX (2025–2029). The objectives of this research project are twofold: firstly, to establish an analytical map of the presence of PFAS in drinking water (tap water, bottled water) in three European cities (Besançon, Bari and Bucharest), and in water discharged from various European industrial sectors (surface treatment, paper mills, laundries), and secondly, to propose innovative solutions for eliminating these substances by biosorption, ion-exchange, and advanced oxidation.

To collect analytical data on the presence of PFAS in tap water, we conducted a local survey to evaluate human exposure to PFAS in 28 private homes in a medium-sized city in France (Besançon), located between Strasbourg and Lyon, two major French metropolises. In this study, we present and discuss the results on the identification and quantification of 65 PFAS substances in 74 residential tap waters, collected in several sampling periods. Concentrations of PFAS were assessed by a French accredited laboratory. To our knowledge, this is the first comprehensive and detailed study investigating the identification and quantification of PFAS in drinking water samples collected in Besançon, France.

## Materials and methods

### Sampling protocol

**Fig. 1:**
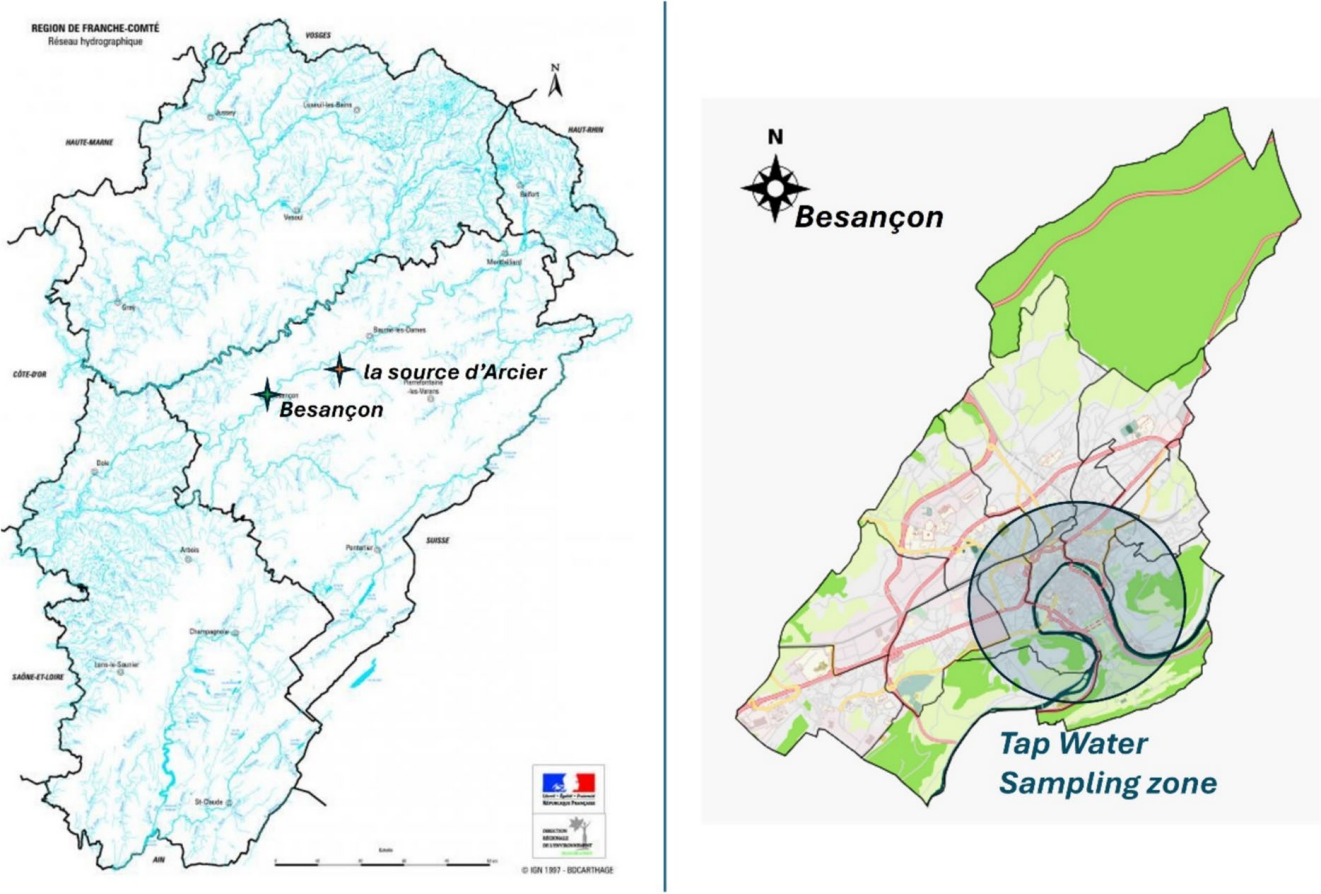
Left: Hydrographic network of the Franche-Comté region showing the location of Besançon and the Arcier spring, the main water supply source for the city. Right: Map of the municipality of Besançon highlighting the urban area and the tap water sampling zone

Tap water comes from the same source (47° 16′ 01″ N, 6° 07′ 16″ E—“*la source d’Arcier*” in Fig. 1), a groundwater that arrives in a reservoir before being treated by filtration and UV disinfection. Tap water was collected from 28 households in Besançon (13 houses and 15 apartments, 47° 14′ 24″ N, 6° 01′ 12″ E), each of which agreed to take part in the study. Only three households use tap water filtration and softening system. Of the 28 households, 19 reported drinking only tap water, 8 both tap and bottled water from eleven different commercial brands, and 1 only bottled water (same brand). In addition to use as a drink, all households use tap water for cooking (except for one), washing, and flushing.

The 28 households were selected on a voluntary basis. These households do not aim to be representative of the entire city, and therefore the results cannot be extrapolated to the city scale (Fig. 1). The aim of the study was to characterize PFAS contamination levels within these selected households.

Tap water sampling protocol, identical for all 28 households, was as follows: All samples were drawn from the kitchen cold water tap as first-draw water, i.e., the first flow of the day, between 06:30 and 07:30; sampling was performed by an accredited service provider to ensure consistency across locations; precautionary measures included thorough handwashing prior to sampling, avoiding contact with the interior of sample bottles and caps, and minimizing atmospheric contamination (*e.g.*, avoiding exposure to cigarette smoke). Samples were collected in PFAS-free high-density polyethylene containers provided by the accredited laboratory. After collection, the samples were stored in a cooler at approximately 5 °C prior to shipment and analysis. In total, 74 tap water samples were collected and analyzed.

### Analysis

All PFAS (65 molecules) and AOF (Adsorbable Organic Fluorine) analyses were carried out by an accredited laboratory, IANESCO (Poitiers, France). IANESCO is a laboratory COFRAC-accredited for the analysis of AOF in wastewater (COFRAC accreditation no. 1–6209, ISO/DIS 18127), the accredited analysis of 16 PFAS including short-chain PFAS in clean water, and 20 PFAS in wastewater with a limit of quantification of 56 ng/L for the Σ_20_ PFAS, the analysis of trifluoroacetic acid (TFA) with a limit of quantification of 50 ng/L on clean water (Table S1, Supplementary Data).

#### PFAS analysis

In this study, 65 PFAS were analyzed by liquid chromatography (LC) with electrospray ionization (ESI) paired with tandem mass spectrometry (MS/MS) using a COFRAC-accredited method. LC–ESI–MS/MS is a sensitive analytical technique that can detect molecules with ultra-short (1–3 carbons), short (C4-C6) and long (C8-C10) chains at trace levels (nanograms per liter) and identify the different types of PFAS in a sample. The list of PFAS analyzed and the limit of quantification of each molecule are given in Table S1 (Supplementary Data).

Samples preparation and clean-up were performed prior to LC–MS/MS analysis. Before extraction, each water sample was acidified to pH 3. Internal standards were then added, and the sample was homogenized by stirring. PFAS were extracted using solid-phase extraction on an anion-exchange column. After column conditioning, 250 mL of sample was loaded onto the column, which was subsequently washed with ultrapure water. Elution was performed in three steps: first with 4 mL of MeOH, followed by two elutions with 4 mL of MeOH containing 0.1% ammonia (v/v), prepared by adding 400 µL of a 25% aqueous ammonia solution per 100 mL of methanol. The extract was then concentrated under vacuum to a final volume of 125 µL, after which 125 µL of methanol was added prior to LC–MS/MS analysis.

The analysis was carried out using an LC20-40 chromatograph coupled with an 8060 triple quadrupole mass spectrometer from Shimadzu. The compounds were separated on an XTERRA C18 chromatographic column (WATERS—50 × 3.0 mm with particles of 3.5 µm), using a MeOH/ACN (acetonitrile)-water phase gradient (flow rate of 0.5 mL/min). The injection volume was 5 µL. Detection was performed using electrospray ionization in negative mode, with two specific MRM transitions monitored for each compound: one used for quantification and the second for confirmation, as reported in Table S2 of the Supplementary Data. For quantification, analytical standards containing each compound and its corresponding internal standards, prepared in mobile-phase solvents, were injected into the analytical system at the beginning of every sample batch (Table S3-S4, Supplementary Data). Then, for each compound, a calibration curve was determined using a quadratic model with a 1/c weighting. The quantification of the samples was then carried out by comparing the response ratio between each compound and its internal standard in the sample to that of the corresponding calibration curve. The internal standards were labeled with carbon-13 (^13^C), as reported in Table S2 of the Supplementary Data.

#### AOF analysis

The AOF index is a measure of the concentration of organic fluorine in a sample. This analysis assesses contamination by PFAS, including those not quantified by conventional analytical methods. This index is interesting because it can account for the total fluorinated compounds (the best-known PFASs and any precursors) that have been adsorbed onto a matrix during preparation of the sample to be analyzed. The methodology is based on an adsorption process coupled with liquid chromatography. It is important to note that TFA is not retained on the adsorbent during sample preparation for AOF determination.

The protocol is the following: A 100 mL volume of diluted or undiluted sample was passed through a series of 2 quartz tubes containing activated carbon specifically designed to trap fluorinated organic compounds; inorganic halides are removed by washing the carbon with a sodium nitrate solution; each carbon tube was calcined in a stream of oxygen at 1050 °C with the addition of water; fluorides released in HF form were trapped by bubbling in a known volume of ultrapure water. The fluoride ions collected were then determined by ion chromatography using an external calibration system. The result is the sum of each column in series, corrected for their respective blanks. The analysis was carried out at least 2 times with 2 different dilutions, and the difference between 2 results must be less than 10% (otherwise, the measurement is repeated). Specifically, the first analysis was carried out with a 1/5th dilution. Depending on the result of that dilution, a new analysis was carried out either with a lower dilution (1/2) or even an analysis of the undiluted sample. If the 1/2 dilution was not usable (case for example of values greater than 200 μg/L), a larger dilution was carried out (1/10 for example), or even several dilutions (1/20; 1/100; 1/500, etc.). The analytical equipment used for AOF was an APU-28 adsorption system (Analytical Jena, France), a combustion system (Analytical Jena, France) and an ion chromatography chain (Metrohm, France).

### Quality assurance and quality control

Quality assurance and quality control (QA/QC) procedures were implemented throughout the analytical workflow to ensure data reliability and traceability (Table S5, Supplementary Data). Calibration blanks, procedural blanks, and control points were included in each analytical sequence to monitor contamination, instrumental stability, and carry-over effects. Blank signals remained below the established acceptance criteria and below the LOQs for the target analytes. Quantification limits and instrument performance were verified daily using calibration standards and quality control injections. Expanded uncertainties for PFAS analyses ranged from 5 to 56%, depending on the compound; when insufficient validation data were available, a conservative uncertainty of 50% was applied. For AOF analyses, combustion efficiency, adsorption recovery, and ion chromatography stability were routinely monitored. In addition, the laboratory regularly participated in interlaboratory comparisons to verify analytical accuracy. Further details regarding QA/QC procedures are provided in the Supplementary Data.

### Ethics statement

Participation of households in this study was voluntary. All participants were informed about the objectives of the study and agreed to the collection and analysis of tap water samples from their homes. The results presented in this work are anonymized and cannot be traced back to individual households. Participating households were informed that the analyses were conducted for research purposes and that the results would be communicated to them individually together with general information on PFAS in drinking water and existing regulatory guidelines. Although the study does not aim to assess compliance of the public water supply, participating households were advised to contact local water authorities if they wished to obtain further information regarding drinking water quality.

## Results and discussion

### PFAS levels in tap water samples from Besançon

Regardless of the water sample analyzed, all AOF index values were below the limit of quantification*, i.e.*, 2 µg/L. In contrast, targeted analyses of 65 PFAS were positive, with 12 molecules possibly quantified in 40 of the 74 tap water samples analyzed. Another notable finding was that TFA was detected and quantified in all water samples, alongside other PFAS present at lower frequencies and concentrations.

#### Common PFAS

For the 74 samples analyzed, only 40 were positive, and 11 of the 65 PFAS substances monitored were identified and quantified (Table 1). Only TFA was systematically detected in all samples. The other 10 substances were: trifluoromethanesulfonic acid (TFMS), 1H,1H,2H,2H-perfluoroctansulfonic acid (H4PFOS) or 6:2-fluorotelomer sulfonic acid (6:2 FTS), perfluoropropanoic acid (PFPrA), perfluorobutanoic acid (PFBA), perfluoropentanoic acid (PFPeA), perfluorohexanoic acid (PFHxA), perfluoroheptanoic acid (PFHpA), perfluorooctanoic acid (PFOA), perfluorobutanesulfonic acid (PFBs), perfluorohexanesulfonic acid (PFHxS), and perfluorooctanesulfonic acid (PFOS).

**Table 1:** Detection frequencies (in %) and concentrations (in ng/L) of 65 PFAS (PFCA = perfluoroalkyl carboxylic acids, PFSA = perfluoroalkane sulfonic acids) monitored in 40 tap water samples from Besançon (Of the 74 samples analyzed, only 40 showed a mixture of PFAS molecules), France (in bold, substances included in the list of 20 regulatory PFAS; for PFCA classification: ultrashort-chain if the number of carbons (C) of the alkyl chain is ≤ 2, short-chain if C is between 3 and 7, or long-chain if C ≥ 7; for PFSA classification: ultrashort-chain if C is 1, 2 or 3, short-chain if C is 4 or 5, or long-chain if C ≥ 6; PFPrA is not included in the table because it was quantified only once)

PFAS name	PFAS abbreviation	PFAS classification (number of carbons)	Number of samples	Frequency	Minimum	Maximum	Mean	Median
Trifluoroacetic acid	TFA	PFCA: ultrashort-chain (C2)	74^a^	100	540	3,805	1,199 ± 722	980
**Perfluorooctanoic acid**	**PFOA**	PFCA: long-chain (C8)	40	90.0	1.2	7.7	2.4 ± 1.7	1.8
**Perfluorooctanesulfonic acid**	**PFOS**	PFSA: long-chain (C8)	40	82.5	1.0	59.0	11.4 ± 15.5	5.2
**Perfluorobutanoic acid**	**PFBA**	PFCA: short-chain (C4)	40	50.0	1.2	6.5	3.1 ± 1.6	2.3
**Perfluorohexanoic acid**	**PFHxA**	PFCA: short-chain (C6)	40	42.5	1.0	19.0	5.7 ± 6.0	2.4
**Perfluoropentanoic acid**	**PFPeA**	PFCA: short-chain (C5)	40	37.5	1.0	32.0	9.6 ± 9.2	5.2
**Perfluoroheptanoic acid**	**PFHpA**	PFCA: long-chain (C7)	40	35.0	1.0	16.0	3.6 ± 3.8	2.7
**Perfluorobutanesulfonic acid**	**PFBs**	PFSA: short-chain (C4)	40	22.5	1.1	4.8	1.8 ± 1.2	1.3
6:2-Fluorotelomer sulfonic acid	H4PFOS	Precursor: long-chain (C8)	40	25.0	1.6	32.0	11.2 ± 10.3	10.0
**Perfluorohexanesulfonic acid**	**PFHxS**	PFSA: long-chain (C6)	40	22.5	1.1	2.2	1.6 ± 0.4	1.6
Trifluoromethanesulfonic acid	TFMS	PFSA: ultrashort-chain (C1)	40	12.5	31.0	97.0	52.2 ± 28.9	34.0
Σ_20_ PFAS^b^			40		1.2	77.7	21.5 ± 22.4	10.6
Σ_65_ PFAS^c^			40		538	3,805	1,119 ± 657	890

^a^only TFA was detected in all the 74 samples analyzed

^b^sum of the 20 regulated PFAS (excluding TFA)

^c^sum of the 65 PFAS analyzed in this study (including TFA)

Of the 20 regulatory substances, 8 were found: PFBA, PFPeA, PFHxA, PFHpA, PFOA, PFBs, PFHxS, and PFOS. In terms of detection frequency (DF), apart from TFA (DF = 100%), which was detected in the 40 samples, the two other substances most frequently found were PFOA and PFOS, both of which are banned in Europe. The following trend was found: TFA (100%) > PFOA (90.0%) > PFOS (82.5%) > PFBA (50.0%) > PFHxA (42.5%) > PFPeA (37.5%) > PFHpA (35.0%) > H4PFOS (25.0%) > PFBs ~ PFHxS (22.5%) >  > TFMS (12.5%) >  > PFPrA (2.5%).

**Fig. 2:**
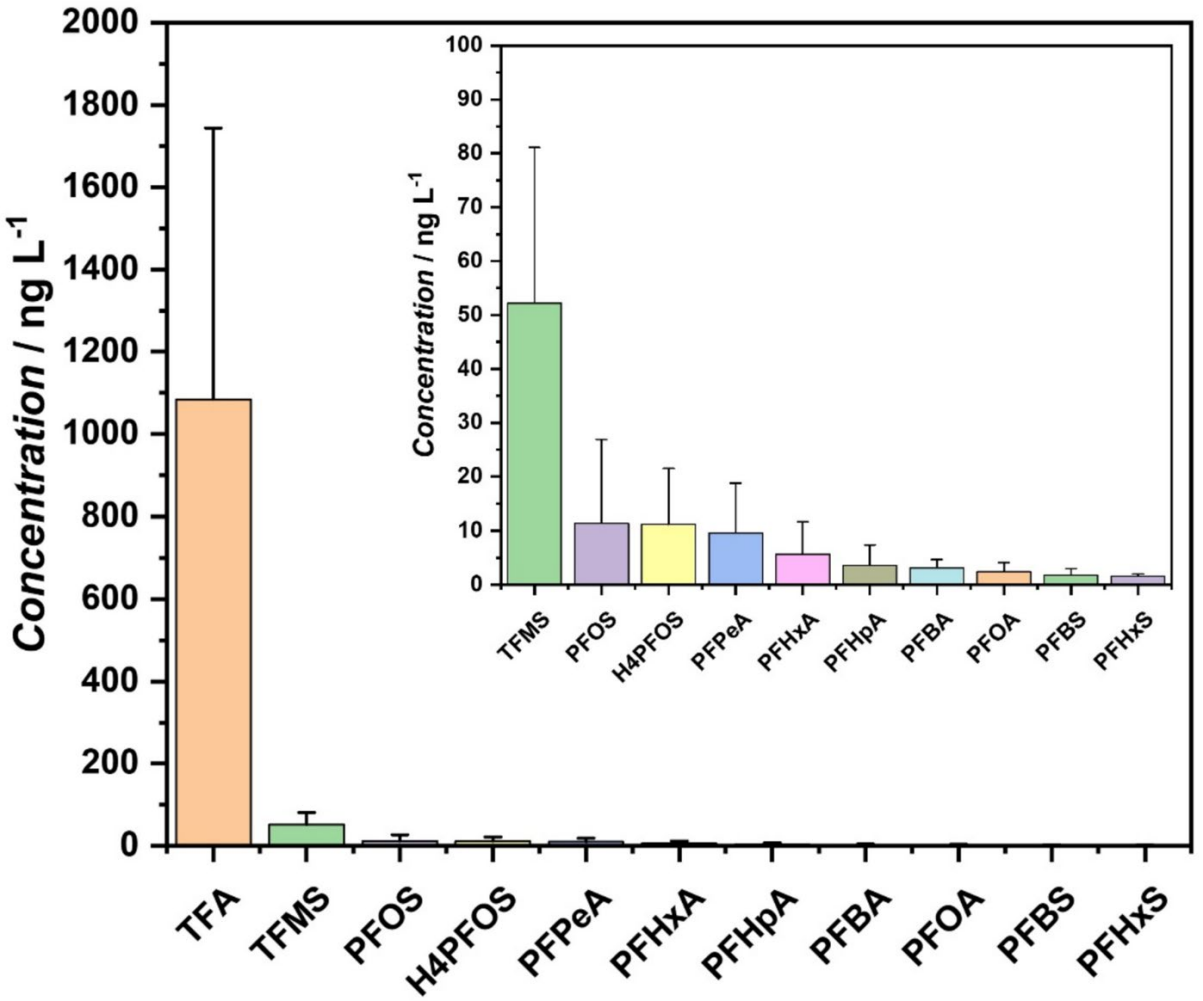
Mean concentrations of eleven perfluoroalkyl substances found in 40 tap water samples (Of the 74 samples analyzed, only 40 showed a mixture of PFAS molecules ranging from 1 to 10, if only the 20 regulatory substances are examined). Error bars represent the standard error of the mean

However, in terms of concentration, Fig. 2 shows that the trend is different, with very short-chain PFASs showing the highest values. The trend is the following for 40 samples: TFA (mean value = 1,084 ng/L) >  > TFMS (52.2 ng/L) > PFOS (11.4 ng/L) > H4PFOS (11.2 ng/L) > PFPeA (9.6 ng/L) > PFHxA (5.7 ng/L) > PFHpA (3.6 ng/L) > PFBA (3.1 ng/L) > PFOA (2.4 ng/L) > PFBs (1.8 ng/L) ~ PFHxS (1.6 ng/L). Zheng et al. [19] reported similar trends for their evaluation levels of ultra-short- and short-chain PFAS in US homes.

When only the 20 regulated substances are considered, concentrations in 40 samples ranged from 1.2 ng/L to 77.7 ng/L, indicating substantial variability. Among the 20 regulatory substances, the highest average concentrations were 11.4 ± 15.5 ng/L, 9.6 ± 9.2, and 5.7 ± 6.0 for PFOS, PFPeA, and PFHxA, respectively. The average concentration for the Σ_20_PFAS was 21.5 ± 22.4 ng/L. Similar values have been reported in the literature (Endirlik et al., 2019; Kaboré et al., 2018; Loos et al., 2007; Park et al., 2018; Schwanz et al., 2016; Shafique et al., 2017; Smalling et al., 2023; Zheng et al., 2023). For those 40 samples that contained a mixture of PFAS, the mean and median concentration for TFA are 1,084 ± 660 ng/L and 845 ng/L, respectively (Table 2). If we consider all 74 samples, the values found are similar (1,096 ± 575 ng/L and 980 ng/L), which shows the reproducibility of the data and the fact that TFA is systematically detected when this molecule is searched even in the absence of other PFAS (Table 2).

**Table 2:** Repeatability of results for three tap water samples analyzed in 4 campaigns (concentrations in ng/L; in bold, substances included in the list of 20 regulatory PFAS: TFA: trifluoroacetic acid; TFMS: trifluoromethanesulfonic acid; H4PFOS: 6:2-fluorotelomer sulfonic acid; PFPrA: perfluoropropanoic acid; PFHxA: perfluorohexanoic acid; PFHpA: perfluoroheptanoic acid; PFBs: perfluorobutanesulfonic acid' PFHxS: perfluorohexanesulfonic acid; PFOA: perfluorooctanoic acid; PFOS: perfluorooctanesulfonic acid; PFPeA: perfluoropentanoic acid)

	Sample 1^a^				Sample 2^a^				Sample 3^a^			
PFAS	Campaign 1	Campaign 2	Campaign 3	Campaign 4	Campaign 1	Campaign 2	Campaign 3	Campaign 4	Campaign 1	Campaign 2	Campaign 3	Campaign 4
TFA	2,000	2,100	920	990	1,100	670	600	560	1,400	1,300	1,100	990
TFMS	< 25	< 25	34	66	< 25	< 25	< 25	< 25	< 25	< 25	33	66
H4PFOS	< 1.0	< 1.0	< 1.0	< 1.0	3.2	17	21	2.0	< 1.0	< 1.0	< 1.0	< 1.0
PFPrA	< 25	< 25	< 25	< 25	< 25	< 25	< 25	< 25	< 25	< 25	< 25	< 25
**PFBA**	4	1.6	2.3	2.1	3.6	5.9	5.5	< 1.0	22	32	28	21
**PFHxA**	1.1	1.0	1.1	1.0	< 1.0	4.8	< 1.0	< 1.0	< 1.0	< 1.0	6.1	1.2
**PFHpA**	2.0	1.0	1.0	1.0	3.4	16	4.4	< 1.0	< 1.0	< 1.0	2.8	< 1.0
**PFBs**	2.4	1.5	1.3	1.2	8.3	< 1	18	1.2	1.7	2.0	1.2	< 1
**PFHxS**	2.0	1.0	1.1	1.1	< 1.0	< 1.0	< 1.0	< 1.0	1.8	1.4	1.6	1.1
**PFOA**	2.5	1.5	1.5	1.4	1.4	1.4	1.2	< 1.0	5.8	4.7	4.5	1.4
**PFOS**	9.0	3.4	5.9	6.1	< 1	< 1	< 1	< 1.0	45.0	39.0	29.0	6.1
**PFPeA**	3.3	1.0	1.6	1.6	13	20	19	1.2	< 1.0	< 1.0	7.4	1.6

^**a**^The three households were chosen to represent different sectors of the distribution network, and the four campaigns correspond to four consecutive months

#### Trifluoroacetic acid

**Fig. 3:**
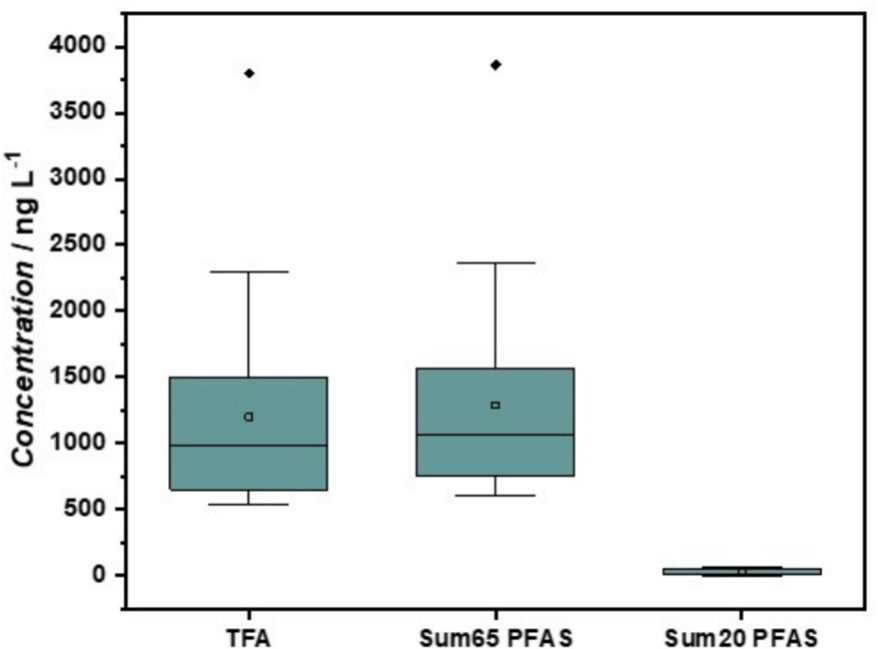
Box plot of the concentrations (in ng/L) of trifluoroacetic acid (TFA), sum of 65 PFAS, and sum of 20 regulated PFAS in tap water. The boxes represent the interquartile range (25th to 75th percentile), the horizontal line inside the box indicates the median, and the square marks the mean. The whiskers extend to the most extreme value within 1.5 times the interquartile range that is not considered an outlier. Diamond-shaped points represent outliers

As reported in Fig. 3, when analyzing a broader range of PFAS, in our case 65 compounds instead of the regulatory 20, the number of substances detected in the 40 samples varies between 2 and 10, with maximum and minimum concentrations of 3,805 ng/L and 538 ng/L, respectively. In this case, the value of the average of the 40 samples is much higher: Σ_65_ PFAS = 1,119 ± 657 ng/L, *i.e.,* around 60 times the value of Σ_20_PFAS 21.5 ± 22.4 ng/L. This high value is explained by the high concentration of TFA, this molecule being included in the list of 65 when it is not in the regulatory list (Table S1).

Many studies on the identification of PFAS in water in general do not include TFA, as this substance is difficult to analyze, with limits of quantification sometime more than 100 ng/L. Nevertheless, when this substance is monitored, the results show that, in the majority of studies, this PFAS is systematically found in much higher concentrations than other PFASs. Indeed, different studies have highlighted the fact that TFA is very present in tap water, with a frequency of detection and levels similar to those found in our study (Albi et al., 2025; Boutonnet et al., 1999; Dvorakova et al., 2023; Endirlik et al., 2019; Sauvé et al., 2023; Sikora et al., 2025; Zhai et al., 2015).

Our results showed that, among the 65 PFAS substances monitored, TFA was systematically detected in all water samples. For tap water (n = 74), the mean concentration of TFA was 1,115 ± 573 ng/L, this value being 11 times higher than the regulatory value (100 ng/L).

Jiao et al., studying the presence of TFA in 39 tap water samples from Shanghai, China, reported that TFA was present in 91% in samples, with concentrations ranging from 1,740 to 7,080 ng/L (Jiao et al., 2023). Other studies also reported that TFA concentrations in drinking water are orders of magnitude much higher than those of other PFAS. For example, TFA is by far the most dominant PFAS in US (Zheng et al., 2023), Turkish (Endirlik et al., 2019), German (Neuwald et al., 2022), and Dutch (Sadia et al., 2023) drinking water.

Numerous studies have also concluded that the presence of TFA in tap water is linked to the contamination of its source (Endirlik et al., 2019), correlated with the degree of urbanization, industrialization and agricultural activity in the catchment area (Joerss et al., 2024; Zhi et al., 2024).

#### Variations

**Fig. 4:**
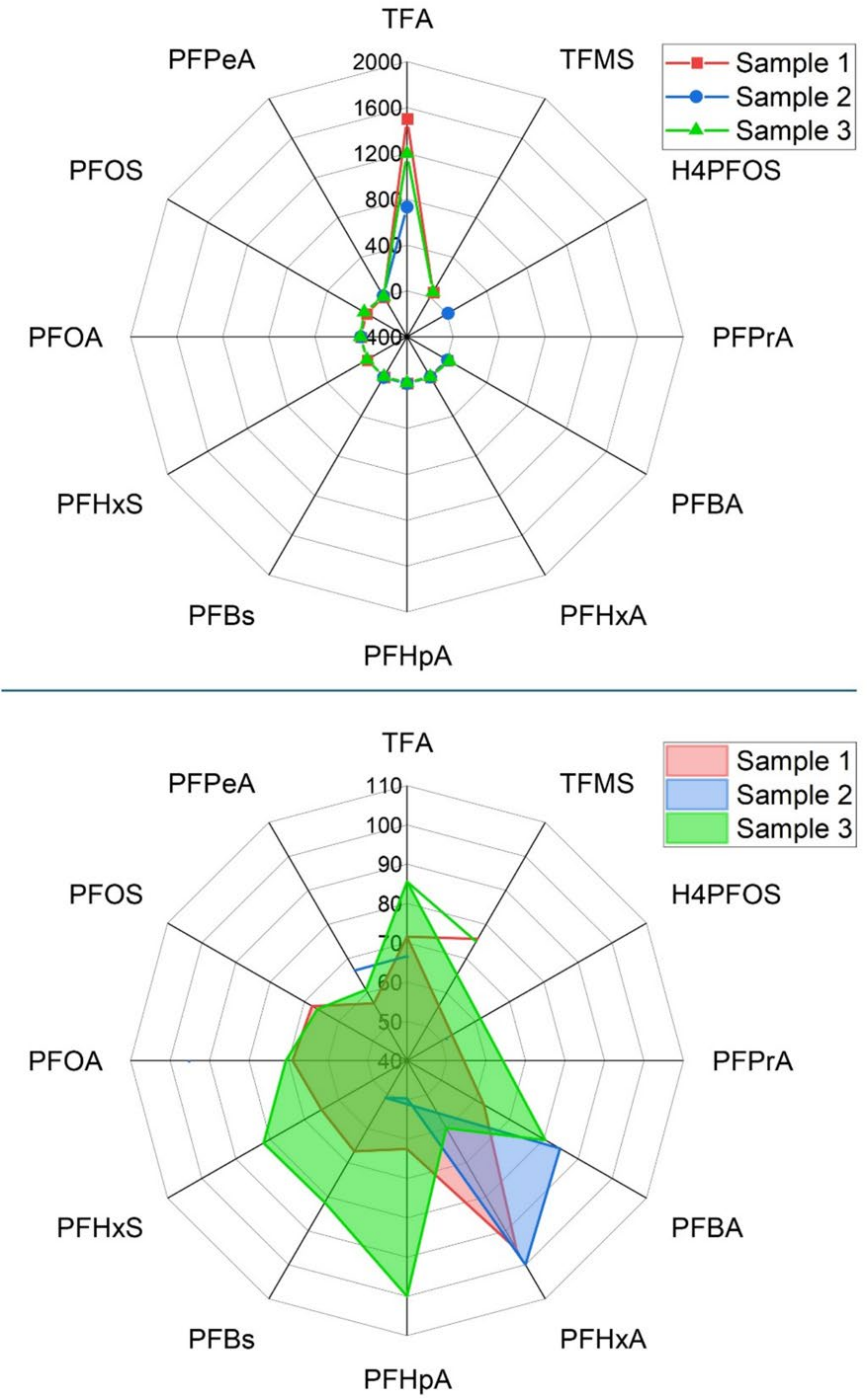
Top: Radar chart of average PFAS concentrations for three households across all four campaigns. Average concentrations of each PFAS compound are plotted for sample 1 (red), sample 2 (blue), and sample 3 (green). The three households were chosen to represent different sectors of the distribution network, and the four campaigns correspond to four consecutive months. Data are shown in actual concentration units (*e.g.*, ng/L), emphasizing absolute dominance of compounds such as trifluoroacetic acid (TFA). Bottom: Normalized radar chart of average PFAS profiles across all campaigns. Values for each PFAS compound have been scaled to 100% based on the maximum concentration observed between the two samples, enabling relative comparison across compounds. Sample 1 is filled in red, sample 2 in blue, and sample 3 in green

Table 2 shows the repeatability of results for three tap water samples analyzed in 4 campaigns under identical conditions. The three households were selected to represent different sectors of the distribution network, and the four campaigns correspond to four consecutive months (one analysis per month). With the exception of TFA concentrations, the levels of the other PFAS were comparable. As shown in Fig. 4, the data also indicate differences in tap water from one household to another, pointing out the uneven distribution of drinking water quality problems (Jaffee, 2024).

TFA dominated the PFAS profile in all samples, indicating a pronounced disparity in absolute concentrations. Sample 1 exhibited higher total PFAS levels, driven primarily by elevated TFA and PFPeA concentrations. To enable a comparative assessment of compound distributions, the data were normalized to the maximum average concentration observed across all samples. This approach revealed notable differences in relative composition: While TFA remained a major component, compounds such as PFBA, PFHxA, and PFHpA were proportionally more abundant in Sample 2. In particular, PFHxA reached near-maximal normalized values in Sample 2, underscoring its relative significance compared to Sample 1. The normalized PFAS profile of Sample 3 reveals a diversified distribution, with several compounds contributing comparably to the overall chemical signature. Unlike samples dominated by a single high-abundance PFAS, Sample 3 displays a balanced composition, with PFHpA, PFHxA, and PFBS emerging as the most influential contributors. Lower relative levels of legacy PFAS such as PFOS and PFOA further highlight a shift toward a broader mixture of short- and mid-chain species. This widespread distribution may indicate diffuse contamination sources or complex transformation processes, underscoring the need for comprehensive monitoring strategies capable of capturing heterogeneous PFAS patterns in environmental matrices.

Collectively, these results suggest that Sample 1 is affected by a point source characterized by high concentrations of a limited subset of PFAS, whereas Sample 2 reflects inputs from more diffuse sources with a broader compound profile, and Sample 3 exhibits an even more diversified and balanced PFAS distribution indicative of widespread, heterogeneous inputs.

For the Σ PFAS, and regardless the number of PFAS compounds detected, the results also showed large disparities between the 28 French households that participated in the study, as the values of the standard deviation that measures the average deviation from the mean are relatively high, particularly for TFA with a value of 1,084 ± 660 ng/L for the 40 samples (Table 2). For the other PFAS, marked variability was also observed. For example, mean concentrations of PFOA and PFPeA were 11.4 ± 15.5 ng/L and 9.6 ± 9.2 ng/L, respectively, reflecting a heterogeneous distribution among samples. However, interpretation of these results remains challenging, as detection frequencies varied widely, ranging from 12.5% to 90% across the 40 samples in which these compounds were detected. In contrast, TFA was consistently detected in all samples analyzed (both the subset of 40 and the full set of 74 samples; Table 2).

Three main hypotheses can be put forward to explain the differences in the concentrations obtained: i) a source water contamination and treatment inefficiency; ii) distribution system effects; and iii) the use of filtration systems by the participants (only three declared having a softener system, an osmosis system or a filtration cartridge installed directly on the kitchen tap).

Table 3 shows the official data on the concentrations of six PFAS systematically found in drinking water distributed to the inhabitants of the city of Besançon. Despite the purification treatment of the source water, the presence of these 6 PFAS in the water before its distribution indicates the contamination of the source water by PFAS. For the same identified molecule, the values are similar, except for PFPeA where the values varied between 8 and 71 ng/L. From one molecule to another, the values also vary, with the highest concentrations being found for PFPeA (24.1 ± 18.6 ng/L) and PFHxA (12.8 ± 7.8 ng/L). The values of standard deviation also confirm a variable efficiency of the water purification treatment. Of the 8 official analyses, only one is higher than the standard set at 100 ng/L, the value of sample 7 being 139 ng/L. These 6 molecules were also quantified in our study. Table 4 also gives the results of the sum of 20 regulatory PFAS. These values are of the same order of magnitude as those described in our study, suggesting that water contamination could come from the production source. Finally, these results, published in the official website of the French minister (Table 4) confirm a contamination of the source and/or a variable efficiency of the water purification treatment.

**Table 3:** Official data published on the website of the French Ministry of Health (source: https://sante.gouv.fr/sante-et-environnement/eaux/eau) on the concentrations of PFAS found in drinking water distributed to the inhabitants of the city of Besançon (8 analyses carried out from December 2024 to February 2025; Σ_20_ PFAS = sum of the 20 regulated substances; Limit: Σ_20_ PFAS < 100 ng/L)

	Sample 1	Sample 2	Sample 3	Sample 4	Sample 5	Sample 6	Sample 7	Sample 8	Sample 9
	December	October	October	October	September	September	August	May	February
**PFBA**	4	5	5	5	9	8	17	9	3
**PFPeA**	16	15	14	18	27	23	71	25	8
**PFHxA**	10	10	9	10	12	14	32	14	4
**PFHPA**	4	3	3	4	6	5	12	6	2
**PFOA**	1	1	1	2	2	2	5	2	< 1
**PFOS**	1	1	2	2	3	2	2	1	< 1
**20 PFAS**	36	35	34	41	59	54	139^a^	57	17

^a^value higher than the standard set at 100 ng/L

**Table 4:** Comparison of the concentrations (range or mean in ng/L) of several PFAS present in tap water samples from various countries (TW = tap water; NS = number of samples; DF = detection frequencies in %; QL = quantification limit; SNS = substance not sought in the analytical method: nd = non-detected)

Country	NS	TFA	TFMS	H4PFOS	PFPrA	PFBA	PFHxA	PFHpA	PFBs	PFHxS	PFOA	PFOS	PFPeA	Reference
France	30	1199 ± 722 DF = 100	29.5 ± 14.9 DF = 16.7	4.7 ± 7.9 DF = 36.7	28.8 ± 21.0 DF = 3.3	2.3 ± 1.7 DF = 53.3	1.2 ± 0.7 DF = 26.7	2.2 ± 2.9 DF = 40.0	3.6 ± 51 DF = 50.0	1.1 ± 0.3 DF = 23.3	2.1 ± 1.2 DF = 90.0	7.2 ± 11.9 DF = 76.7	5.3 ± 7.8 DF = 50.0	This study
Turkey	94	SNS				0.27–1.93 DF = 57	0.08–2.90 DF = 94	0.08–1.65 DF = 64	0.11–0.85 DF = 87	0.10–2.18 DF = 64	0.10–2.37 DF = 55	0.09–2.04 DF = 55	0.08–1.23 DF = 74	(Endirlik et al., 2019)
France	27	SNS	SNS	SNS	SNS	SNS	5.8–6.8	13.0–33.0	2.0–15.0	< QL	8.7–18.0	11.9–3.0	SNS	(Schwanz et al., 2016)
Brazil	30	SNS	SNS	SNS	SNS	SNS	< QL-15.9	5.2–36	0.18–16	< QL	3.0–46.0	4.6–44.0	SNS	(Schwanz et al., 2016)
Canada	59	SNS	SNS	SNS	SNS	2.3	0.59	0.33	0.24	0.30	0.67	1.0	0.44	(Kaboré et al., 2018)
US		230	5.58		nd	nd			nd					(Liang et al., 2023)
US		450	3.20		< 5	nd			nd					(Liang et al., 2023)
US		428	< 2.5		< 5	nd			nd					(Liang et al., 2023)
US	22					3.4–24.0		3.1–4.5		5.0–5.2	6.8–13.6	9.4–17.8	3.7–10.0	(Von Behren et al., 2024)
South Korean	44						5.5 ± 7.7 DF = 86		2.6 ± 1.7 DF = 77	15.1 ± 33.9 DF = 82	5.8 ± 8.7 DF = 95	0.7 ± 0.3 DF = 61	5.5 ± 5.5 DF = 64	(Park et al., 2018)
Spain		SNS									0.32–6.28	0.39–0.87		(Ericson et al., 2008)
Spain	39	SNS	SNS	SNS	SNS	SNS	14.0–58.0	4.1–42.0	2.8–24.0	< QL	3.8–29.0	2.0–140.0	SNS	(Schwanz et al., 2016)
Germany		SNS				0.54–0.57	0.91–0.93	0.16–0.21	1.23–1.30	0.04–0.06	5.80–6.50		0.51–0.59	(Shafique et al., 2017)
China	39	1174–7080 DF = 91												(Jiao et al., 2023)
China	9	1712 ± 74 DF = 100												(Dong et al., 2023)
China		155^a^												(Zhai et al., 2015)
Italy	6	SNS						0.3–0.8 0.5 ± 0.2			1.0–2.9 2.4 ± 0.7	6.2–9.7 8.1 ± 1.2		(Loos et al., 2007)
Italy		SNS									500	30		(Mastrantonio et al., 2018)
Brazil		SNS								0.15–1.00	0.35–2.82	0.58–6.70		(Quinete et al., 2009)
Germany	46	900^a^ DF = 90												(Neuwald et al., 2022)
Czech Republic	27						2.01	0.95	0.34				1.86	(Kozisek et al., 2025)
US		41–150^a^												(Boutonnet et al., 1999)
The Netherlands	11	351 ± 305 DF = 100												(Sadia et al., 2023)
Sweden		SNS								25		45		(Gyllenhammar et al., 2015)
US	81	< QL-210 DF = 74	SNS	< QL-0.57 DF = 11	< QL-19 DF = 99	< QL-7.8 DF = 84	< QL-6.1 DF = 83	< QL-1.2 DF = 79	< QL-0.16 DF = 85	< QL-1.1 DF = 99	< QL-3.6 DF = 99	< QL-1.6 DF = 99	< QL-7.7 DF = 25	(Zheng et al., 2023)
The Netherlands	7	245 ± 167 DF = 100												(Sadia et al., 2023)

Although the tap water supplied to all households in this study originates from the same source, concentrations found in individual homes differed substantially. Despite this fact, the concentrations found in homes are different. These differences can also be explained by the type and state of deterioration of the pipes of the water distribution network, and that of buildings and houses (for example, those using plastic). However, it is difficult to establish a possible correlation without further measurements and information.

Data published show that PFAS contamination of tap water can be related to contamination in its source correlated with the degree of urbanization/industrialization in the catchment area (Chow et al., 2021; Dvorakova et al., 2023; Endirlik et al., 2019; Eschauzier et al., 2013; Jaffee, 2024; Li et al., 2019; Liang et al., 2023; Neuwald et al., 2022; Park et al., 2018; Sadia et al., 2023; Zhai et al., 2015; Zhi et al., 2024). Zhai et al. concluded that PFAS contamination levels in Chinese drinking water were in relation with the presence of anthropogenic activities (Zhai et al., 2015). Li et al. also reported that, in China, PFAS pollution levels in drinking water are positively influenced by the existence of industrial sources close to water resources and by the different city levels and population density (pollution in medium sized city > big city > town) (Li et al., 2019). Endirlik et al. studied in detail the PFAS levels in drinking water samples from Turkey and concluded that contamination of tap water was related to contamination in its source (Endirlik et al., 2019). Neuwald et al. indicated that PFAS can be introduced into the water cycle both through industrial processes, agricultural activities, and by the transformation of pharmaceutical substances present in domestic discharges or hydrofluorocarbon refrigerants into the atmosphere, which can then reach the aqueous environment via atmospheric deposition (Neuwald et al., 2022).

Kozisek et al. investigated the presence of 28 PFAS in the drinking water supplies in the Czech Republic. Tap water samples (n = 27) from sources near potentially contaminated areas such as chemical and car industry sites, airports, and ski resorts were contaminated, with concentrations and compositions varying across different water sources. Total PFAS concentrations varied from undetectable to 90.8 ng/L, with PFPeA, PFHxA, PFHpA and PFBS being the most abundant, detected in over 70% of samples. The authors concluded on the need for enhanced surveillance to also address hidden or non-obvious sources of contamination, in order to better identify the source(s) of contamination of the resource (Kozisek et al., 2025).

### Regulatory framework for drinking water

In Europe, the revised 2020 Drinking Water Directive requires all member states to monitor a list of 20 PFAS of concern or considered as priority substances, from January 2026 (EU Directive, 2020/2184 of 16 December 2020). However, this list of 20 priority PFAS does not include TFA (Cordner et al., 2024).

The EU directive also imposes a regulatory threshold not to be exceeded of 100 ng/L for the sum of these 20 substances (“PFAS Sum”), and/or a total amount of measurable PFAS of 500 ng/L (“PFAS Total”) representing all PFAS in water intended for human consumption.

TFA is the subject of regulatory uncertainty, and numerous discussions are underway to classify this molecule as a priority substance. Unlike the two best-studied and best-known PFAS substances, PFOA and PFOS, which have been banned in Europe, there is still a lack of chemical and toxicological data on TFA (Cordner et al., 2024; EU Directive, 2020/2184 of 16 December 2020). Only recently, in March 2026, through Directive (EU) 2026/805 concerning surface waters, TFA is expected to be included in the sum of 25 PFAS with a threshold value of 100 ng/L. Furthermore, TFA, which has so far not been regulated, is now included in the group of 25 PFAS and will become subject to assessment for groundwater in the next review. In addition to TFA, the substances that distinguish the list of 25 PFAS in the Surface Water Directive (2026/805) from the 20 PFAS listed in the Drinking Water Directive (2020/2184) are: PFTeDA, HFPO-DA (commonly known as GenX), ADONA, and 6:2 FTS. It is noteworthy that, even under this recent regulatory framework, TFA concentrations observed in this study largely exceed this threshold (EU Directive, 2026/805 of 30 March 2026). However, it should be emphasized that, for drinking water intended for human consumption, no specific regulatory limit value for TFA has yet been established.

Today, neither the European Union (EU) nor the World Health Organization (WHO) have set a binding limit value for TFA. Only Denmark has set a binding and health-based limit value for TFA in drinking water at 9,000 ng/L. The German Federal Environment Agency recently proposed a drinking water health guidance value for TFA of 60,000 ng/L (Neuwald et al., 2022). In addition, Italy, through Legislative Decree 102/2025, has established a limit value for TFA of 10,000 ng/L. However, the mandatory monitoring requirement for TFA will only come into force in January 2027 (*Decreto Legislativo* n. 102/2025, Italy, 19 June 2025). The French Agency for Food, Environmental and Occupational Health & Safety (ANSES) also recommended the same value of 60,000 ng/L, with the aim in the coming years to reduce it to 10,000 ng/L (*Bulletin Officiel Santé Protection Sociale Solidarité* n° 2025/4).

Our results show that, out of 74 tap water samples, 40 have concentrations of 12 PFAS, with the average value for the sum of the 20 priority molecules below the regulatory threshold of 100 ng/L. The highest concentration was 77.7 ng/L if the sum of 20 is taken into account, while the value is 3,805 ng/L for the sum of 65. Regardless of the number of substances, the concentrations of the two PFAS most frequently found, PFOS and PFOA, are below the 100 ng/L threshold. Two other PFAS molecules, PFPeA and PFPrA (not included in the list of 20) have significant concentrations, but are within the regulatory limit.

### Data comparison

The comparison of PFAS concentrations reported in the literature (Table 4) shows substantial variability across studies, reflecting differences in analytical approaches, water sources, and regional contamination patterns (Adewuyi & Li, 2024). Despite these differences, several consistent trends emerge. PFAS are detected in tap waters worldwide, and particular attention is required for TFA, which appears frequently across studies (Boiteux et al., 2012; Boutonnet et al., 1999; Dong et al., 2023; Dvorakova et al., 2023; Ericson et al., 2008; Gyllenhammar et al., 2015; Igarashi et al., 2021; Jiao et al., 2023; Joerss et al., 2024; Llorca et al., 2012; Mastrantonio et al., 2018; Quinete et al., 2009; Sauvé et al., 2023). Ultrashort-chain PFAS (≤ 3 carbons) are often predominant both in the number of detected substances and in their concentrations (Bai & Son, 2021; Dong et al., 2023; Kaboré et al., 2018). PFOS and PFOA, although banned in Europe, are still detected in many works but at variable levels (Adewuyi & Li, 2024; Eschauzier et al., 2013; Von Behren et al., 2024). Other PFAS such as PFBS, PFHxA, PFPeA, and TFA are also commonly reported, in agreement with our findings (Boettger et al., 2025; Kozisek et al., 2025). In most cases, the sum of the 20 regulated PFAS remains below the 100 ng/L limit defined by the European Directive for drinking water. Notably, TFA appears to be a special case: it is detected in almost all types of water matrices, including tap water, bottled water, groundwater, surface water, and industrial effluents, underscoring its widespread occurrence and the importance of including it in future monitoring efforts.

Our results showed that 40 tap water samples had a mixture of PFAS molecules ranging from 1 to 10 with an average concentration for the Σ_20_PFAS of 21.5 ± 22.4 ng/L. Among the 20 regulatory substances, the highest average concentrations were 11.4 ± 15.5 ng/L, 9.6 ± 9.2, and 5.7 ± 6.0 for PFOS, PFPeA, and PFHxa, respectively. Similar values have been reported in the literature (Loos et al., 2007; Schwanz et al., 2016; Shafique et al., 2017; Smalling et al., 2023; Spyrakis & Dragani, 2023; Zheng et al., 2023). For example, during analysis of tap water from Leipzig, Germany, Shafique et al. reported that PFOA (6.2 ng/L) was the major PFAS found among eight other molecules and covered 53% of load (Σ_16_PFAS = 11.5 ng/L)(Shafique et al., 2017).

Kaboré et al. reported that 59 Canadian tap water samples showed notable differences in the concentrations of 29 PFAS (TFA was not monitored), depending mainly on their source, with detection frequencies and concentrations comparable to those found in our work (Kaboré et al., 2018). Park et al., evaluating the contamination status of 44 South Korean tap water samples, also reported similar mean and median to those found in French water samples (Park et al., 2018). These authors indicated that the differences in concentrations can be attributed to the contamination of the source by industrial activities (Park et al., 2018). In their evaluation of the current contamination status of 10 PFAS in Turkish 94 tap water samples, Endirlik et al. found a total PFAS concentration ranged from 0.08 to 11.27 ng/L (Σ_10_ PFAS). The maximum PFOA (DF = 55%) and PFOS (55%) levels were 2.37 and 2.04 ng/L, respectively. The other PFAS frequently detected with higher concentration were PFHxA (DF = 94), PFBS (87), and PFPeA (74) (Endirlik et al., 2019). Tan et al. recently assessed PFAS levels in Singapore’s drinking water: detected values ranged from 0.30 ng/L to 15.17 ng/L. The authors concluded that these low PFAS levels indicated minimal health risks (Tan et al., 2025). Ji et al. reported higher and more variable values for Chinese tap water: The concentration for the sum of PFAS ranged from 45.2 to 155.6 ng/L, the main PFAS found were PFOA, PFBS, PFHxA, and PFBA (Ji et al., 2025).

### Risk communication and advice for households

The households participating in this study were informed that the analyses were conducted for research purposes and that the results do not constitute a regulatory assessment of drinking water compliance. All results presented in this study are anonymized and cannot be linked to individual households.

Individual analytical results were communicated to the participating households together with general information regarding PFAS in drinking water and the current European regulatory framework. Particular attention was given to explaining that the concentrations of the regulated PFAS substances remained below the current European regulatory limit for the sum of 20 PFAS in drinking water.

At the same time, the systematic detection of other PFAS compounds, particularly trifluoroacetic acid, highlights the importance of continued monitoring and ongoing scientific evaluation of these substances. Participants were informed that toxicological knowledge and regulatory approaches for some PFAS are still evolving.

Households wishing to further limit potential exposure to PFAS were advised that mitigation strategies reported in the literature may include the use of certified activated carbon filtration systems, ion exchange resins, or reverse osmosis treatment, which have been shown to reduce PFAS concentrations in drinking water under certain conditions (Crini et al., 2026) or the seeking of additional information from local water authorities regarding drinking water quality. These recommendations were provided for general informational purposes only.

It is important to emphasize that the aim of this work is to inform both participating households and the scientific and regulatory communities as well as water management authorities about the potential need for a revised water monitoring policy that would include additional PFAS, particularly trifluoroacetic TFA, in routine assessments. Such an approach could contribute to a better characterization of human exposure to these compounds and support the establishment of appropriate guideline values for drinking water. Furthermore, improved knowledge of the occurrence of these pollutants may help water quality monitoring programs identify potential sources of contamination and contribute to strategies aimed at reducing human exposure to PFAS.

## Conclusions

The aim of this study was to measure the adsorbable organic fluorine (AOF) index and to quantify per- and polyfluoroalkyl substances (PFAS) as emerging drinking water contaminants in tap water collected from 74 samples obtained in 28 households in Besançon, France. All analyses were performed by an accredited laboratory under the French Ministry of Health.

Overall, the AOF index was below the limit of quantification (2 µg/L) in all samples. In contrast, targeted PFAS analysis (65 compounds) revealed the presence of multiple substances, with 12 compounds quantified in 40 of the 74 samples. These included TFA, TFMS, H4PFOS, PFPrA, PFBA, PFPeA, PFHxA, PFHpA, PFOA, PFBS, PFHxS, and PFOS. Multiple PFAS co-occurred in individual samples, with mixtures ranging from 2 to 10 compounds per sample.

Among the investigated substances, TFA was the most frequently detected compound, occurring in all samples and showing the highest overall prevalence. The cumulative PFAS concentrations (sum of 65 compounds) varied considerably between samples, ranging from 538 to 3,805 ng/L, highlighting strong spatial variability. In contrast, the sum of the 20 regulated PFAS remained low (1.2–77.7 ng/L), remaining below the regulatory threshold of 100 ng/L.

TFA was consistently the dominant compound in all samples and was present at concentrations substantially higher than those of regulated PFAS such as PFOA and PFOS, which were detected at lower levels and within regulatory limits.

In conclusion, this study demonstrates that TFA is a ubiquitous contaminant in tap water in Besançon, despite not being included in current regulatory PFAS lists. Its widespread occurrence and dominance over regulated PFAS highlight the need to broaden monitoring strategies beyond the currently regulated compounds. Future work should investigate spatial variability across a larger number of households and assess TFA levels at different stages of the water supply chain, including source water and post-treatment conditions.

## Supplementary Information

Below is the link to the electronic supplementary material.

Supplementary file1 (DOCX 42 KB)

## Data Availability

Not applicable.
