# Supplementary material for: Identification and quantification of PFAS in the tap water of a French City, Besançon
Source: Environ Monit Assess. 2026 May 23;198(6):640. doi: 10.1007/s10661-026-15483-y (PMC13198494; doi:10.1007/s10661-026-15483-y)
Supplement: Supplementary file 1 — Supplementary file1 (DOCX 42 KB) [file 10661_2026_15483_MOESM1_ESM.docx]

**Identification and quantification of PFAS in the tap water of a French city, Besançon**

**Grégorio Crini^1,*^, Chiara Mongioví^2^, Corina Bradu^3^, Nicolas Audonnet^4^, Stephen Yquel^4^, Dario Lacalamita^1,5,*^**

^1^ Laboratoire Chrono-environnement, Université Marie et Louis Pasteur, 16 route de Gray, 25000 Besançon, France

^2^ Istituto per i Processi Chimico-Fisici, CNR, via E. Orabona 4, Bari, 70125, Italy

^3^ Department of Systems Ecology and Sustainability, PROTMED Research Centre, University of Bucharest, Bucharest, Romania

^4^ Laboratoire IANESCO, 6 rue Carol Heitz, 86000 Poitiers, France

^5^ Department of Chemistry, Università degli Studi di Bari Aldo Moro, via E. Orabona 4, Bari, 70125, Italy

**Further details regarding QA/QC**

Further details regarding quality assurance and quality control (QA/QC) procedures are provided below.

For each analytical sequence, calibration blanks were injected at the beginning and end of the run to verify the absence of contamination and monitor instrumental stability. For all analytes, blank signals remained below 10% of the response of the corresponding quantification standard. For TFA, calibration blank signals were consistently below 10% of the signal measured for the lowest calibration standard, which was prepared at a concentration four times lower than the limit of quantification (LOQ).

Procedural blanks were prepared daily during sample preparation using the same PFAS-free bottles, extraction devices, reagents, and analytical workflow as those applied to environmental samples. Blank concentrations were required to remain below the method LOQs. TFA blank concentrations were generally <15 ng/L, while PFAS procedural blanks were typically below detection limits for all analytes except 6:2 FTS, for which occasional low-level signals remained below the LOQ.

Quantification limits and instrumental sensitivity were verified daily through calibration sequences including the lowest calibration standard. Instrument performance was assessed using signal intensity, signal-to-noise ratio, calibration linearity, and sequence stability criteria. Analytical runs not meeting acceptance criteria were repeated following instrument maintenance.

Expanded uncertainties for PFAS analyses were estimated from repeated validation experiments performed under varying analytical conditions. Depending on the analyte, expanded uncertainties ranged from 5% to 56%. For compounds lacking sufficient validation data, a conservative expanded uncertainty of 50% was applied.

Specific QA/QC procedures were also implemented for adsorbable organic fluorine (AOF) analyses. Instrument blanks were included in each analytical sequence and were required to remain below 1 µg/L. Combustion efficiency was verified daily by introducing quartz fiber paper spiked with 2 µg AOF directly into the combustion furnace. Adsorption efficiency was monitored using ultrapure water spiked at 20 µg/L AOF and processed as a routine sample. Ion chromatography stability was evaluated through periodic injections of control standards, including a control point at the LOQ at the end of each analytical sequence.

In addition, the accredited laboratory participated in interlaboratory comparison exercises at least twice per year to verify analytical accuracy and reproducibility.

**TABLES**

**Table S1.** List of PFAS analyzed with the limit of quantification (LOQ) values (LOQ in µg/L; in bold, the 20 PFAS include in the regulatory list; the sum of these 20 substances must be less than or equal to 100 ng/L)

|  | **Acronym(s)** | **Full name** | **LOQ** |
| --- | --- | --- | --- |
| **1** | **PFBA** | **perfluorobutanoic acid** | **0.001** |
| **2** | **PFDA** | **perfluorodecanoic acid** | **0.001** |
| **3** | **PFDoDA** | **perfluorododecanoic acid** | **0.005** |
| **4** | **PFHpA** | **perfluoroheptanoic acid** | **0.001** |
| **5** | **PFHxA** | **perfluorohexanoic acid** | **0.001** |
| **6** | **PFNA** | **perfluorononanoic acid** | **0.001** |
| **7** | **PFOA** | **perfluorooctanoic acid** | **0.001** |
| **8** | **PFPeA** | **perfluoropentanoic acid** | **0.001** |
| **9** | **PFTrDA** | **perfluorotridecanoic acid** | **0.005** |
| **10** | **PFUnDA** | **perfluoroundecanoic acid** | **0.005** |
| **11** | **PFBs** | **perfluorobutane sulfonic acid** | **0.001** |
| **12** | **PFDoDS** | **perfluorododecane sulfonic acid** | **0.005** |
| **13** | **PFDS** | **perfluorodecane sulfonic acid** | **0.005** |
| **14** | **PFHpS** | **perfluoroheptane sulfonic acid** | **0.005** |
| **15** | **PFHxS** | **perfluorohexane sulfonic acid** | **0.001** |
| **16** | **PFNS** | **perfluorononane sulfonic acid** | **0.005** |
| **17** | **PFOS** | **perfluorooctane sulfonic acid** | **0.001** |
| **18** | **PFPeS** | **perfluoropentane sulfonic acid** | **0.001** |
| **19** | **PFTriS / PFTrDS** | **perfluorotridecane sulfonic acid** | **0.005** |
| **20** | **PFUDS** | **perfluoroundecane sulfonic acid** | **0.005** |
| 21 | TFA | trifluoroacetic acid | 0.050 |
| 22 | TIMeS | triflic acid | 0.025 |
| 23 | PFPrA | perfluoropropanoic acid | 0.025 |
| 24 | 3:3 FTCA | 2H,2H,3H,3H-perfluorohexanoic acid | 0.001 |
| 25 | 5:3 FTCA | 2H,2H,3H,3H-perfluorooctanoic acid | 0.001 |
| 26 | 8:2 FTCA / FOEA / 8:2 FTA | 2H,2H-perfluorodecanoic acid | 0.001 |
| 27 | 7:3 FTCA | 2H,2H-perfluorooctanoic acid | 0.001 |
| 28 | FOUAE / FOUEA / 8:2 FTUCA | 2H-perfluoro-2-decenoic acid | 0.001 |
| 29 | 62-FTOH / 6:2 FTOH | 2-perfluorohexyl ethanol (6:2) | 0.005 |
| 30 | 6:2 FTAB / CAPSTONE B | 6:2 fluorotelomer sulfonamide betaine | 0.005 |
| 31 | 82-FTOH / 8:2 FTOH | 2-perfluorooctyle ethanol (8:2) | 0.100 |
| 32 | 7HPFHpA | 7H-dodecafluoroheptanoic acid | 0.001 |
| 33 | 9Cl-PF3ONS | perfluoro-2-((6-chlorohexyl)oxy)ethane sulfonic acid | 0.001 |
| 34 | 10:2 FTS | perfluorododecanesulfonic acid | 0.001 |
| 35 | FDEA / 10:2 FTCA | 2H,2H-perfluorododecanoic acid | 0.001 |
| 36 | FHUEA / 6:2 FTUCA | 2H-perfluoro-2-octenoic acid | 0.001 |
| 37 | N-EtFOSE | N-ethyl-N-(2-hydroxyethyl)perfluorooctane sulfonamide | 0.001 |
| 38 | N-MEFOSE | N-(2-hydroxyethyl)-N-methylperfluorooctane sulfonamide | 0.001 |
| 39 | PFeCHS | decafluoro(pentafluoroethyl)cyclohexane sulfonate | 0.001 |
| 40 | PFEESA | perfluoro(2-ethoxyethane) sulfonic acid | 0.001 |
| 41 | NFDHA | perfluoro-3,6-dioxaheptanoic acid | 0.001 |
| 42 | PFMPA | perfluoro-3-methoxypropanoic acid | 0.005 |
| 43 | PFMBA | perfluoro-4-methoxybutanoic acid | 0.001 |
| 44 | ADONA | 4,8-dioxa-3H-perfluorononanoic acid | 0.001 |
| 45 | 11Cl-PF3OUDS | 11-chloroperfluoro-3-oxaundecanesulfonic acid | 0.001 |
| 46 | APF36DO | perfluoro-3,6-dioxaoctanoate ammonium | 0.001 |
| 47 | 3,7-DMPFOA/P37DMOA | perfluoro-3,7-dimethyloctanoic acid | 0.001 |
| 48 | C6O4 | perfluoro((5-methoxy-1,3-dioxolan-4-yl)oxy) acetic acid | 0.005 |
| 49 | GenX | perfluoro-2-propoxypropanoic acid | 0.010 |
| 50 | 8: 2 / H4PFDS | 1H,1H,2H,2H-perfluorodecane sulfonate | 0.001 |
| 51 | 4:2 FTS / H4PFHS | 1H,1H,2H,2H-perfluorohexane sulfonate | 0.001 |
| 52 | 6:2 FTS / H4PFOS | 1H,1H,2H,2H-perfluoctyl sulfonate | 0.001 |
| 53 | 6:2 FTCA | 2H,2H-perfluoroctanoic acid | 0.001 |
| 54 | MEFOSA | N-methyl perfluorooctane sulfonamide | 0.001 |
| 55 | MEFOSAA | 2-(N-methylperfluorooctanesulfonamido) acetic acid | 0.001 |
| 56 | NEtFOSA | 2-(N-ethylperfluorooctanesulfonamido) acetic acid | 0.001 |
| 57 | NEtFOSAA | 2-(N-ethylperfluorooctanesulfonamido) acetic acid | 0.001 |
| 58 | PFBSA | perfluoro-1-butane sulfonamide | 0.005 |
| 59 | PFHxDA | perflurohexadecanoic acid | 0.010 |
| 60 | PFHXSA | perfluorohexane sulfonamide | 0.001 |
| 61 | PFODA | perflurooctadecanoic acid | 0.010 |
| 62 | PFOSA / FOSA | perfluorooctane sulfonamide | 0.001 |
| 63 | PFPRS | perfluoropropanesulfonic acid | 0.001 |
| 64 | PFTeDA | perfluorotetradecanoic acid | 0.005 |
| 65 | PMPA | perfluoro-2-(perfluoromethoxy)propanoic acid | 0.001 |

**Table S2.** Overview of all PFAS included in the targeted analysis, reporting monitored MRM transitions, retention times (RT), corresponding internal standards (ISTD), calibration ranges, and limits of quantification (LOQ).

| **Molecules** | **1^st^ Transition** | **2^nd^ Transition** | **RT (min)** | **ISTD** | **Calibration** | **LOQ (µg/L)** |
| --- | --- | --- | --- | --- | --- | --- |
| Trifluoroacetic acid (TFA) | 113.10 > 69.00 | 113.10 > 19.15 | 3,2 | ^13^C_1__TFA | 2.5 - 500 µg/L | 0,05 |
| Triflic acid (TIMeS) | 149.10 > 79.95 | 149.10 > 98.90 | 2,0 | / | 2.5 - 500 µg/L | 0,025 |
| PFPrA (perfluoropropanoic acid) | 163.10 > 119.05 | 163.10 > 68.95 | 2,8 | / | 2.5 - 500 µg/L | 0,025 |
| PFBA (perfluorobutanoic acid) | 213.00 > 168.90 | 213.00 > 19.10 | 3,1 | ^13^C_3__PFBA | 1.0 - 50 µg/L | 0,001 |
| PFBS (perfluorobutanesulfonic acid) | 298.90 > 79.90 | 298.90 > 98.90 | 5,7 | ^13^C_4__PFBs | 1.0 - 50 µg/L | 0,001 |
| PFDA (perfluorodecanoic acid) | 512.90 > 468.80 | 512.90 > 218.90 | 8,6 | ^13^C_9__PFDA | 1.0 - 50 µg/L | 0,001 |
| PFHpA (perfluoroheptanoic acid) | 362.90 > 318.90 | 362.90 > 168.95 | 7,0 | ^13^C_7__PFHpA | 1.0 - 50 µg/L | 0,001 |
| PFHxA (perfluorohexanoic acid) | 312.90 > 268.90 | 312.90 > 118.95 | 6,2 | ^13^C_6__PFHxA | 1.0 - 50 µg/L | 0,001 |
| PFHxS (perfluorohexanesulfonic acid) | 398.90 > 98.90 | 398.90 > 79.90 | 7,2 | ^13^C_6__PFHxS | 1.0 - 50 µg/L | 0,001 |
| PFNA (perfluorononanoic acid) | 462.90 > 418.90 | 462.90 > 218.90 | 8,1 | ^13^C_9__PFNA | 1.0 - 50 µg/L | 0,001 |
| PFOA (perfluorooctanoic acid) | 412.90 > 368.90 | 412.90 > 168.95 | 7,6 | ^13^C_8__PFOA | 1.0 - 50 µg/L | 0,001 |
| PFOS (perfluorooctanesulfonic acid) | 498.85 > 79.95 | 498.85 > 98.80 | 8,2 | ^13^C_8__PFOS | 1.0 - 50 µg/L | 0,001 |
| PFPeA (perfluoropentanoic acid) | 262.70 > 218.95 | 262.70 > 19.00 | 5,1 | ^13^C_5__PFPeA | 1.0 - 50 µg/L | 0,001 |
| PFOSA (perfluorooctanesulfonamide) | 497.90 > 78.05 | 497.90 > 305.00 | 10,7 | ^13^C_8__PFOSA | 1.0 - 50 µg/L | 0,001 |
| PFDS (perfluorodecanesulfonic acid) | 598.80 > 79.85 | 598.80 > 168.85 | 9,0 | ^13^C_12__PFDoA | 1.0 - 50 µg/L | 0,005 |
| 6:2 FTS (H4PFOS; 6:2 fluorotelomer sulfonate) | 426.85 > 406.90 | 426.85 > 80.80 | 7,6 | ^13^C_2__6:2 FTS-D4 | 1.0 - 50 µg/L | 0,001 |
| PFUnDA (perfluoroundecanoic acid) | 562.75 > 518.90 | 562.75 > 269.00 | 9,0 | ^13^C_9__PFUA | 1.0 - 50 µg/L | 0,005 |
| PFDoDA (perfluorododecanoic acid) | 612.75 > 568.85 | 612.75 > 269.10 | 9,3 | ^13^C_12__PFDoA | 1.0 - 50 µg/L | 0,005 |
| PFTrDA (perfluorotridecanoic acid) | 662.75 > 618.95 | 662.75 > 369.00 | 9,6 | ^13^C_12__PFDoA | 1.0 - 50 µg/L | 0,005 |
| PFPeS (perfluoropentanesulfonic acid) | 348.70 > 79.90 | 348.70 > 118.85 | 6,6 | ^13^C_6__PFHxS | 1.0 - 50 µg/L | 0,001 |
| PFHpS (perfluoroheptanesulfonic acid) | 448.55 > 79.80 | 448.55 > 98.90 | 7,8 | ^13^C_6__PFHxS | 1.0 - 50 µg/L | 0,005 |
| PFNS (perfluorononanesulfonic acid) | 548.60 > 79.95 | 548.60 > 230.00 | 8,7 | ^13^C_8__PFOS | 1.0 - 50 µg/L | 0,005 |
| PFUnDS (perfluoroundecanesulfonic acid) | 648.60 > 230.00 | 648.60 > 79.85 | 9,3 | ^13^C_12__PFDoA | 1.0 - 50 µg/L | 0,005 |
| PFDoDS (perfluorododecanesulfonic acid) | 698.60 > 79.90 | 698.60 > 280.00 | 9,6 | ^13^C_6__PFTeDA | 1.0 - 50 µg/L | 0,005 |
| PFTrDS / PFTriS (perfluorotridecanesulfonic acid) | 748.60 > 79.90 | 748.60 > 380.00 | 9,8 | ^13^C_6__PFTeDA | 1.0 - 50 µg/L | 0,005 |
| PFHxDA (perfluorohexadecanoic acid) | 812.65 > 768.95 | 812.65 > 169.05 | 10,2 | ^13^C_6__PFTeDA | 1.0 - 50 µg/L | 0,01 |
| PFODA (perfluorooctadecanoic acid) | 912.65 > 418.90 | 912.65 > 868.85 | 10,5 | ^13^C_6__PFTeDA | 1.0 - 50 µg/L | 0,01 |
| PFTeDA (perfluorotetradecanoic acid) | 712.65 > 668.95 | 712.65 > 318.90 | 9,8 | ^13^C_6__PFTeDA | 1.0 - 50 µg/L | 0,005 |
| GenX (hexafluoropropylene oxide dimer acid, HFPO-DA) | 329.05 > 169.05 | 329.05 > 285.05 | 6,5 | ^13^C_6__PFHxA | 1.0 - 50 µg/L | 0,01 |
| ADONA (4,8-dioxa-3H-perfluorononanoic acid) | 377.00 > 251.00 | 377.00 > 85.00 | 7,1 | ^13^C_8__PFOA | 1.0 - 50 µg/L | 0,001 |
| C6O4 (perfluoro([5-methoxy-1,3-dioxolan-4-yl]oxy)acetic acid) | 338.95 > 112.95 | 338.95 > 84.95 | 6,8 | ^13^C_8__PFOA | 1.0 - 50 µg/L | 0,005 |
| 4:2 FTS (H4PFHS; 4:2 fluorotelomer sulfonate) | 327.15 > 306.90 | 327.15 > 81.05 | 5,8 | ^13^C_2__4:2 FTS-D4 | 1.0 - 50 µg/L | 0,001 |
| 8:2 FTS (H4PFDS; 8:2 fluorotelomer sulfonate) | 527.05 > 507.00 | 527.05 > 81.00 | 8,0 | ^13^C_2__8:2 FTS-D4 | 1.0 - 50 µg/L | 0,001 |
| PFBSA (perfluorobutanesulfonamide) | 299.10 > 78.00 | 297.50 > 219.00 | 7,0 | ^13^C_3__PFBA | 1.0 - 50 µg/L | 0,001 |
| 6:2 FTAB (Capstone B; 6:2 fluorotelomer sulfonamide betaine) | 569.10 > 549.05 | 569.10 > 446.10 | 7,8 | ^13^C_8__PFOA | 1.0 - 50 µg/L | 0,05 |
| APF36DO (ammonium perfluoro-3,6-dioxaoctanoate) | 251.00 > 135.05 | 251.00 > 69.15 | 6,6 | ^13^C_3__PFBA | 1.0 - 50 µg/L | 0,001 |
| 7H-PFHpA (7H-perfluoroheptanoic acid) | 345.00 > 281.15 |  | 6,1 | ^13^C_3__PFBA | 1.0 - 50 µg/L | 0,001 |
| 9Cl-PF3ONS (perfluoro(2-((6-chlorohexyl)oxy)ethanesulfonic acid) | 530.90 > 350.95 | 532.90 > 352.90 | 9,4 | ^13^C_3__PFBA | 1.0 - 50 µg/L | 0,001 |
| PFPrS (perfluoropropanesulfonic acid) | 249.10 > 80.00 | 249.10 > 99.00 | 4,7 | ^13^C_5__PFPeA | 1.0 - 50 µg/L | 0,001 |
| PFHxSA (perfluorohexanesulfonamide) | 398.95 > 78.05 | 398.95 > 79.10 | 8,9 | ^13^C_6__PFHxS | 1.0 - 50 µg/L | 0,001 |
| PMPA (perfluoro-2-(perfluoromethoxy)propanoic acid) | 228.95 > 185.05 | 228.95 > 85.05 | 3,5 | ^13^C_3__PFBA | 1.0 - 50 µg/L | 0,001 |
| 3,7-DMPFOA (perfluoro-3,7-dimethyloctanoic acid) | 513.00 > 468.90 | 513.00 > 269.00 | 8,4 | ^13^C_3__PFBA | 1.0 - 50 µg/L | 0,001 |
| FDEA (2H,2H-perfluorododecanoic acid; 10:2 FTCA) | 577.00 > 493.10 | 577.00 > 63.00 | 8,6 | ^13^C_8__PFOA | 1.0 - 50 µg/L | 0,001 |
| 10:2 FTS (perfluorododecanesulfonic acid) | 626.95 > 606.90 | 626.95 > 81.15 | 9,4 | ^13^C_9__PFUA | 1.0 - 50 µg/L | 0,001 |
| 11Cl-PF3OUDS (chlorinated perfluoroether sulfonic acid) | 630.90 > 451.05 | 632.90 > 453.00 | 10,8 | ^13^C_6__PFTeDA | 1.0 - 50 µg/L | 0,001 |
| 3:3 FTCA (2H,2H,3H,3H-perfluorohexanoic acid) | 241.00 > 77.00 | 241.00 > 117.00 | 4,0 | ^13^C_5__PFPeA | 1.0 - 50 µg/L | 0,001 |
| 5:3 FTCA (2H,2H,3H,3H-perfluorooctanoic acid) | 341.00 > 237.10 | 341.00 > 217.10 | 6,3 | ^13^C_8__PFOA | 1.0 - 50 µg/L | 0,001 |
| 6:2 FTCA (fluorotelomer carboxylic acid) | 377.00 > 293.10 | 377.00 > 63.00 | 6,3 | ^13^C_8__PFOA | 1.0 - 50 µg/L | 0,001 |
| FHUEA / 6:2 FTUCA (2H-perfluoro-2-octenoic acid) | 357.00 > 293.10 | 357.00 > 243.10 | 6,3 | ^13^C_8__PFOA | 1.0 - 50 µg/L | 0,001 |
| 7:3 FTCA (2H,2H-perfluorooctanoic acid) | 441.00 > 317.00 | 441.00 > 337.00 | 7,5 | ^13^C_8__PFOA | 1.0 - 50 µg/L | 0,001 |
| 8:2 FTCA / FOEA / 8:2 FTA (2H,2H-perfluorodecanoic acid) | 477.00 > 392.90 | 477.00 > 63.20 | 7,3 | ^13^C_8__PFOA | 1.0 - 50 µg/L | 0,001 |
| FOUAE / FOUEA / 8:2 FTUCA (2H-perfluoro-2-decenoic acid) | 456.95 > 393.00 |  | 7,4 | ^13^C_8__PFOA | 1.0 - 50 µg/L | 0,001 |
| FOSA (perfluorooctanesulfonamide) | 497.90 > 78.05 | 497.90 > 305.00 | 10,7 | ^13^C_8__PFOSA | 1.0 - 50 µg/L | 0,001 |
| MeFOSA (N-methylperfluorooctanesulfonamide) | 511.95 > 169.05 | 511.95 > 291.00 | 13,2 | ^13^C_6__PFTeDA | 1.0 - 50 µg/L | 0,001 |
| MeFOSAA (N-methylperfluorooctanesulfonamidoacetic acid) | 569.95 > 418.95 | 569.95 > 482.95 | 8,3 | ei_NMeFOSAA-D3 | 1.0 - 50 µg/L | 0,001 |
| NEtFOSAA (N-ethylperfluorooctanesulfonamidoacetic acid) | 584.00 > 418.95 | 584.00 > 526.00 | 8,6 | ei_NEtFOSAA-D5 | 1.0 - 50 µg/L | 0,001 |
| N-EtFOSE (N-ethyl-N-(2-hydroxyethyl)perfluorooctanesulfonamide) | 630.00 > 59.00 |  | 13,5 | ^13^C_6__PFTeDA | 1.0 - 50 µg/L | 0,001 |
| NFDHA (perfluoro-3,6-dioxaheptanoic acid) | 294.95 > 201.00 | 294.95 > 85.00 | 5,9 | ^13^C_7__PFHpA | 1.0 - 50 µg/L | 0,001 |
| N-MeFOSE (N-methyl-N-(2-hydroxyethyl)perfluorooctanesulfonamide) | 616.00 > 59.00 |  | 12,9 | ^13^C_6__PFTeDA | 1.0 - 50 µg/L | 0,001 |
| PFeCHS (decafluoro(pentafluoroethyl)cyclohexanesulfonate) | 460.70 > 381.05 | 460.70 > 119.00 | 8,1 | ^13^C_6__PFHxS | 1.0 - 50 µg/L | 0,001 |
| PFEESA (perfluoro(2-ethoxyethane)sulfonic acid) | 314.95 > 135.00 | 314.95 > 82.95 | 6,5 | ^13^C_6__PFHxS | 1.0 - 50 µg/L | 0,001 |
| PFMBA (perfluoro-4-methoxybutanoic acid) | 278.95 > 85.00 |  | 5,4 | ^13^C_5__PFPeA | 1.0 - 50 µg/L | 0,001 |
| PFMPA (perfluoro-3-methoxypropanoic acid) | 228.95 > 85.00 | 228.95 > 185.05 | 3,5 | ^13^C_3__PFBA | 1.0 - 50 µg/L | 0,001 |
| NEtFOSA (N-ethylperfluorooctanesulfonamide) | 526.00 > 169.05 | 526.00 > 219.10 | 13,8 | ^13^C_6__PFTeDA | 1.0 - 50 µg/L | 0,001 |

**Table S3.** Calibration range and preparation details for TFA and short‑chain PFAS (Method 655), including compound identifiers (ID), theoretical concentrations (Cth), and preparation steps for each calibration level (SMei: Internal standard stock solution; SM calibration: Primary stock standard solution; SF calibration: Further diluted standard solution; UPW: Ultrapure water).

| **TFA calibration range (Method 655) and short-chain compounds** | | | | | | |
| --- | --- | --- | --- | --- | --- | --- |
| ID | C_th_ (µg/L) | Preparation | | | | |
| SMei | 1000 | Stock internal standard solution |  |  |  |  |
| SM calibration | 100 000 | 10 mg of powder, diluted to 100 mL in MeOH | | | | |
| SF calibration | 1 000 | 0.1 mL of SM calibration solution, diluted to 10 mL in MeOH | | | | |
| std0 | 0 | 0 mL SF calibration + 50 mL UPW + 1 mL SMei, diluted to 100 mL in ACN | | | | |
| std1 | 2,5 | 0.025 mL SF calibration + 5 mL UPW + 0.1 mL SMei, diluted to 10 mL in ACN | | | | |
| std2 | 5 | 0.050 mL SF calibration + 5 mL UPW + 0.1 mL SMei, diluted to 10 mL in ACN | | | | |
| std3 | 10 | 0.1 mL SF calibration + 5 mL UPW + 0.1 mL SMei, diluted to 10 mL in ACN | | | | |
| std4 | 20 | 0.2 mL SF calibration + 5 mL UPW + 0.1 mL SMei, diluted to 10 mL in ACN | | | | |
| std5 | 50 | 0.5 mL SF calibration + 5 mL UPW + 0.1 mL SMei, diluted to 10 mL in ACN | | | | |
| std6 | 100 | 1 mL SF calibration + 5 mL UPW + 0.1 mL SMei, diluted to 10 mL in ACN | | | | |
| std7 | 200 | 0.020 mL SM calibration + 5 mL UPW + 0.1 mL SMei, diluted to 10 mL in ACN | | | | |
| std8 | 500 | 0.050 mL SM calibration + 5 mL UPW + 0.1 mL SMei, diluted to 10 mL in ACN | | | | |

**Table S4.** Calibration range and preparation details for PFAS (Method 503), including compound identifiers (ID), theoretical concentrations (Cth), and preparation steps for each calibration level (SMei: Internal standard stock solution; SM calibration: Primary stock standard solution; SF calibration: Further diluted standard solution; UPW: Ultrapure water).

| **PFAS calibration range (Method 503)** | | | | | | |
| --- | --- | --- | --- | --- | --- | --- |
| ID | C_th_ (µg/L) |  | | | | |
| Scei | 1 000 | Internal standard stock solution |  |  |  |  |
| SM calibration | 100 000 | 10 mg of powder, diluted to 100 mL in MeOH | | | | |
| SF calibration | 100 | Prepared from SM stock: 20 µL of SM (50 mg/L) or 10 µL of SM (100 mg/L) or 500 µL of SM (2 mg/L), diluted to 10 mL in MeOH | | | | |
| std0 | 0 | 0 mL SF calibration + 50 mL UPW + 0.25 mL Scei, diluted to 100 mL in MeOH | | | | |
| std1 | 1 | 0.1 mL SF calibration + 5 mL UPW + 0.025 mL Scei, diluted to 10 mL in MeOH | | | | |
| std2 | 2,5 | 0.25 mL SF calibration + 5 mL UPW + 0.025 mL Scei, diluted to 10 mL in MeOH | | | | |
| std3 | 5 | 0.5 mL SF calibration + 5 mL UPW + 0.025 mL Scei, diluted to 10 mL in MeOH | | | | |
| std4 | 10 | 1 mL SF calibration + 5 mL UPW + 0.025 mL Scei, diluted to 10 mL in MeOH | | | | |
| std5 | 20 | 2 mL SF calibration + 5 mL UPW + 0.025 mL Scei, diluted to 10 mL in MeOH | | | | |
| std6 | 50 | 5 mL SF calibration + 5 mL UPW + 0.025 mL Scei, diluted to 10 mL in MeOH | | | | |

**Table S5.** Analytical performance data for 23 PFAS compounds determined by LC-MS/MS. Values include mean relative deviation (RDT), mean coefficient of variation (CV), population size (n), calculated, verified, and retained limits of quantification (LOQ, µg/L), and expanded uncertainty (U%)

| **Molecule** | **Mean RDT (%)** | **Mean CV (%)** | **Population (n)** | **Calculated LOQ (µg/L)** | **Verified LOQ (µg/L)** | **Retained LOQ (µg/L)** | **Expanded Uncertainty U (%)** |
| --- | --- | --- | --- | --- | --- | --- | --- |
| **H4PFOS** | 103 | 7.0 | 68 | 0.010 | 0.01 | 0.01 | 25 |
| **PFBA** | 96 | 12.0 | 72 | 0.010 | 0.01 | 0.01 | 30 |
| **PFBS** | 89 | 16.9 | 72 | 0.010 | 0.01 | 0.01 | 38 |
| **PFDA** | 97 | 6.0 | 72 | 0.010 | 0.01 | 0.01 | 25 |
| **PFDODA** | 103 | 11.2 | 72 | 0.010 | 0.01 | 0.01 | 27 |
| **PFDoS** | 69 | 21.3 | 53 | 0.010 | 0.01 | 0.01 | 46 |
| **PFDS** | 87 | 24.9 | 57 | 0.050 | 0.05 | 0.05 | 53 |
| **PFHpA** | 96 | 4.6 | 72 | 0.010 | 0.01 | 0.01 | 25 |
| **PFHpS** | 77 | 16.3 | 72 | 0.010 | 0.01 | 0.01 | 36 |
| **PFHxA** | 101 | 8.7 | 72 | 0.010 | 0.01 | 0.01 | 25 |
| **PFHxDA** | 69 | 25.6 | 50 | 0.050 | 0.05 | 0.05 | 56 |
| **PFHxS** | 94 | 11.2 | 72 | 0.010 | 0.01 | 0.01 | 28 |
| **PFNA** | 94 | 4.8 | 72 | 0.010 | 0.01 | 0.01 | 5 |
| **PFNS** | 47 | 18.6 | 71 | 0.010 | 0.01 | 0.01 | 41 |
| **PFOA** | 98 | 4.3 | 72 | 0.010 | 0.01 | 0.01 | 25 |
| **PFOS** | 94 | 13.8 | 71 | 0.010 | 0.01 | 0.01 | 36 |
| **PFPeA** | 99 | 12.9 | 71 | 0.010 | 0.01 | 0.01 | 30 |
| **PFPeS** | 84 | 12.0 | 72 | 0.010 | 0.01 | 0.01 | 28 |
| **PFTeDA** | 95 | 12.1 | 54 | 0.050 | 0.05 | 0.05 | 31 |
| **PFTrDA** | 95 | 19.8 | 52 | 0.050 | 0.05 | 0.05 | 43 |
| **PFTrDS** | 69 | 26.2 | 51 | 0.050 | 0.05 | 0.05 | 56 |
| **PFUDS** | 68 | 23.1 | 52 | 0.050 | 0.05 | 0.05 | 52 |
| **PFUNDA** | 98 | 8.4 | 72 | 0.010 | 0.01 | 0.01 | 25 |
